# Supplementary material for: The role of MEGF10 in myoblast fusion and hypertrophic response to overload of skeletal muscle
Source: J Muscle Res Cell Motil. 2025 Jan 18;46(4):275–92. doi: 10.1007/s10974-024-09686-4 (PMC13751773; doi:10.1007/s10974-024-09686-4)
Supplement: Supplementary file 1 [file 10974_2024_9686_MOESM1_ESM.pdf]

In vivo and in vitro analyses of MEGF10 function demonstrate its role in myoblast fusion and hypertrophic response to overload of skeletal muscle

Louise Richardson, Ruth Hughes, Colin A Johnson, Stuart Egginton, Michelle Peckham

Supplementary Material

Supplementary Tables

Supplemental Table 1: Primary Antibodies used:

Antibody	Source	Species	Dilution (IF)	Dilution (WB)
A4.1025 (anti-sarcomeric myosin)	ATCC hybridoma	Mouse	1:10	N/A
c-myc (9E10)	Sigma	Mouse	N/A	1:5000
GFP (AB10145)	Millipore	Rabbit	N/A	1:1000
Pax7 supernatant.	DSHB (hybridoma)	Mouse	1:20	N/A
MyoD (MA1-41017)	Thermo Scientific	Mouse	1:100	1:500
MyoD (C-20)	Santa Cruz (sc-304)	Rabbit	1:2000	N/A
Myogenin (F5D)	DSHB	Mouse	1:50	1:1000
Myogenin (MA511486)	Invitrogen	Mouse	1:50	N/A
Laminin L9393	Sigma	Rabbit	1:30	N/A
DAPI 40043	Sigma	N/A	1:500	N/A

Supplemental Table 2. Genotypic ratio of mice born from Megf10+/- matings. Total number of WT, heterozygous and homozygous mice and corresponding ratio. A Chi-squared test reveals that the genotypic ratio is not significantly different from that expected (1:2:1). however, we had fewer homozygous mice for our experiments.

	**WT**	**Heterozygous**	**Homozygous**
**Total**	13	20	6
**Ratio**	2	3	1

Supplementary figures

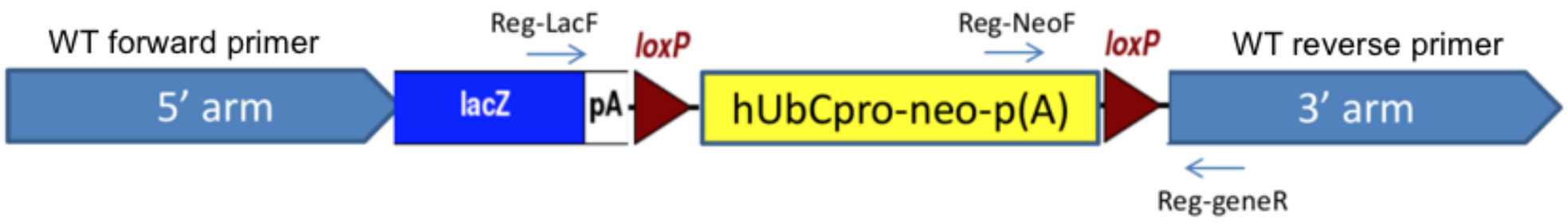

Supplementary Fig. 1. Primer strategy for TaqMan assay for genotyping mice. Schematic showing forward and reverse primer, human ubiquitin C gene promoter (hUbCpro-neo-p(A)) selection cassette flanked by loxP sites and lacZ sequence within the mutant (tm1) allele. Primers used in genotyping used sequence from lacZ (Reg-LacF) and the neomycin selection cassette (tm1a allele: Reg Neo-F), the reverse primer Reg-geneR. The readout of LAC Z+ WT+ = heterozygous, LAC Z+ WT- = homozygous, and LAC Z- WT+ = wild-type.

Sequences of the genotype primers were: LAC-Z: Forward primer: CGATCGTAATCACCCGAGTGT; Reverse primer: CCGTGGCCTGACTCATTCC, Reporter 1: CCAGCGACCAGATGAT; Reporter 2: N/A

And for MEGF10-1 WT; Forward primer: CTACCGGACAGCCTACCG; Reverse primer: CTTTCATAAAATCCTGGGCAACACT; Reporter 1: TATAGACGCAAATCCC, Reporter 2: N/A

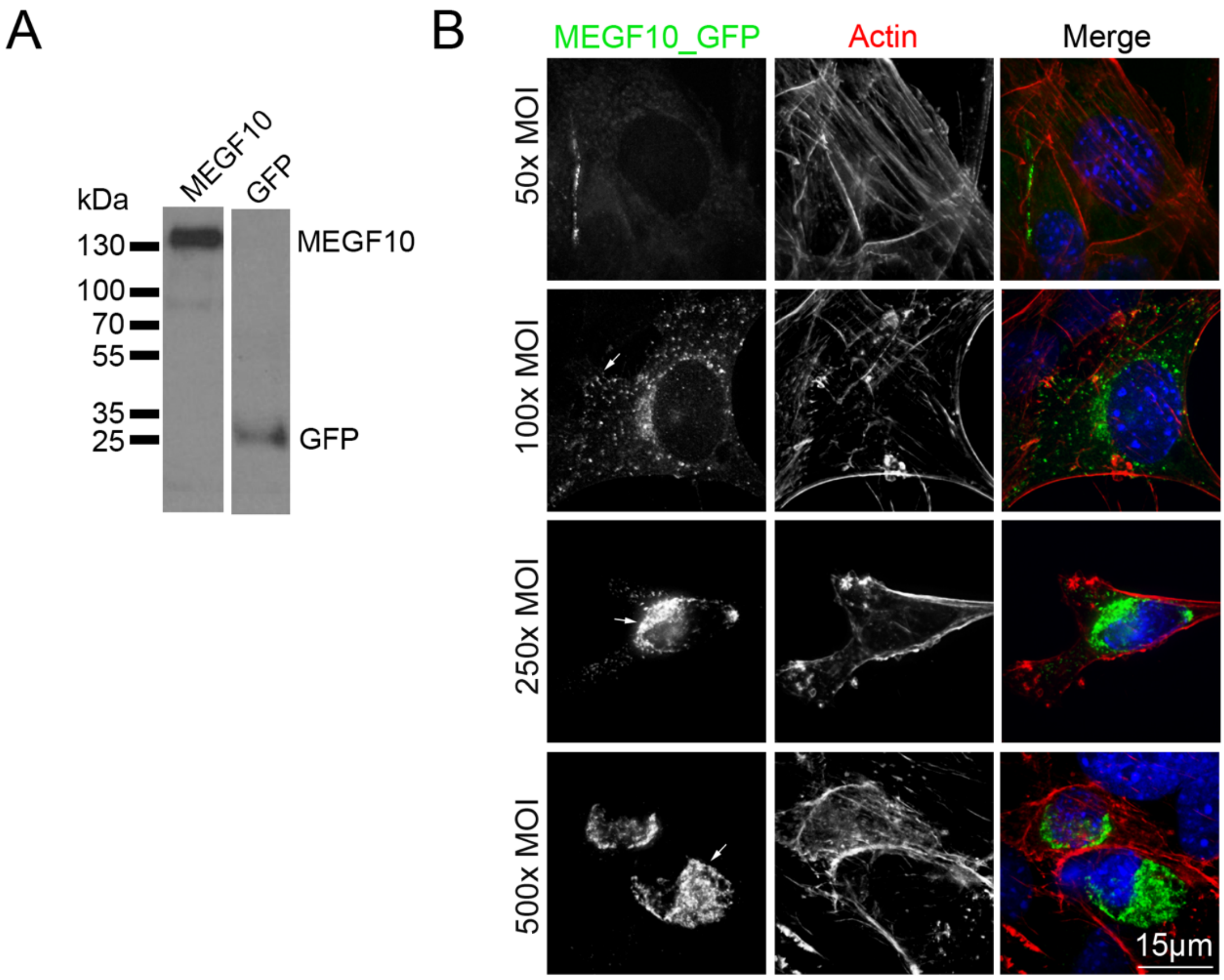

Supplemental Fig. 2: **eGFP and eGFP-MEGF10 expression tests.** **A**: anti-GFP blots for MEGF10-eGFP and the GFP adenovirus expressed in cells. An MOI of 100 was used. **B:** Localisation of MEGF10-EGFP at different MOIs. At an MOI of 50, very little signal is observed. At an MOI of 100, MEGF10-eGFP is found in vesicles and at the plasma membrane (arrowed). At MOIs of 250 and 500, most of the MEGF10-EGFP is in the Golgi (arrowed)

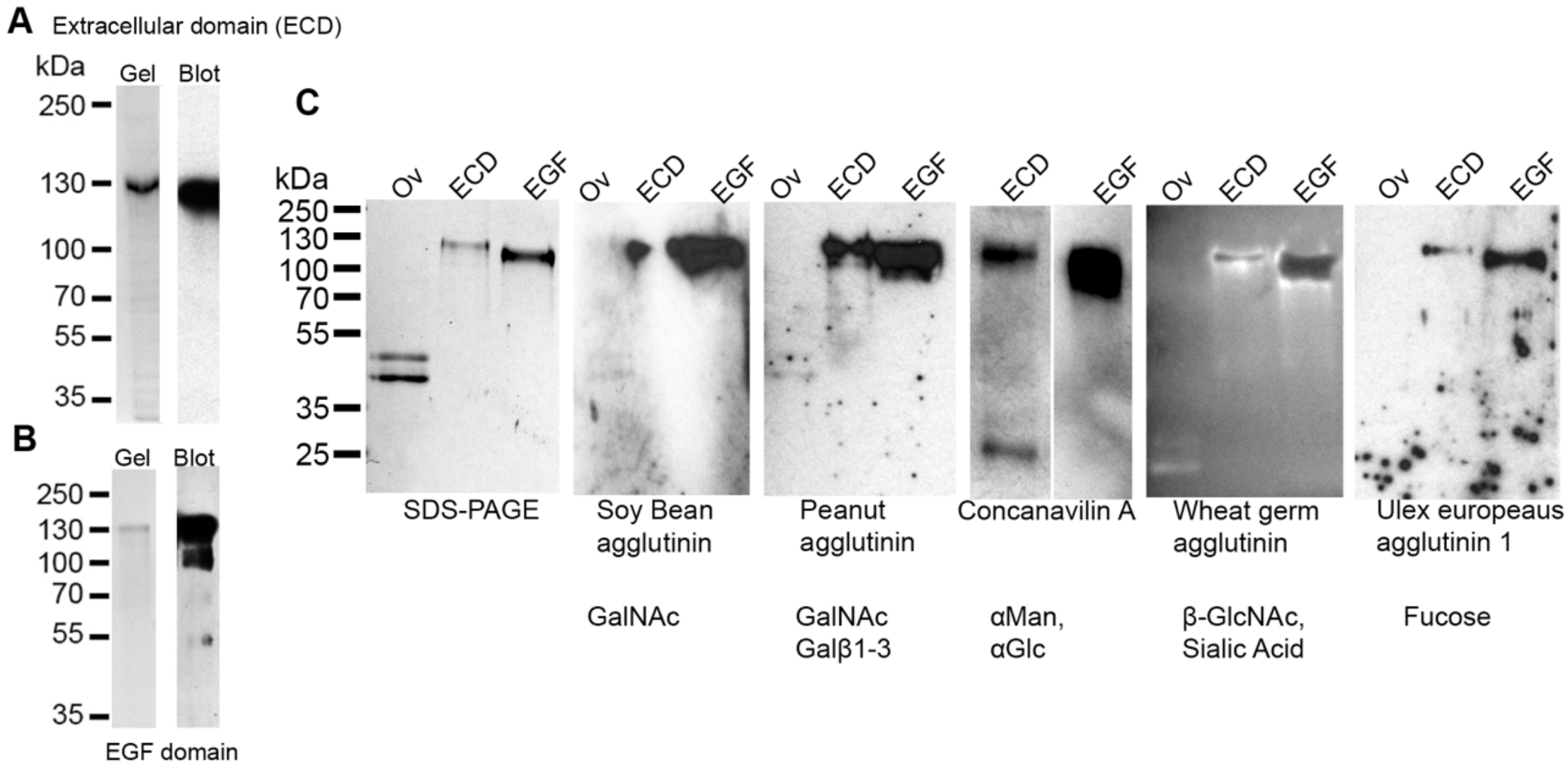

Supplemental Fig 3: Expression, purification and Lectin blots for ECD and EGF. A and B: Gels and blots (anti-Myc Tag) for purified extracellular domain (ECD) (**A**) and EGF domains (**B**) of MEGF10 to show the purified protein and any contaminants. **C**: SDS Page gels of Ovalbumin (Ov), ECD and EGF domains together with lectin blots using each of the specific lectins, and what they recognise, as shown.
